# Regulatory T Cells in Early Life: Comparative Study of CD4^+^CD25^high^ T Cells from Foals and Adult Horses

**DOI:** 10.1371/journal.pone.0120661

**Published:** 2015-03-19

**Authors:** Eman Hamza, Jelena Mirkovitch, Falko Steinbach, Eliane Marti

**Affiliations:** 1 Department of Clinical Research and Veterinary Public Health, Vetsuisse Faculty, University of Bern, Bern, Switzerland; 2 School of Veterinary Medicine, Faculty of Health and Medical Sciences, University of Surrey, Guildford, United Kingdom; Wayne State University, UNITED STATES

## Abstract

The immune system of mammals is subject to continuous development during the postnatal phase of life. Studies following the longitudinal development of the immune system in healthy children are limited both by ethical considerations and sample volumes. Horses represent a particular valuable large animal model for T regulatory (Treg) cells and allergy research. We have recently characterised Treg cells from horses, demonstrated their regulatory capability and showed both their expansion and induction *in vitro*. Insect bite hypersensitivity (IBH) is a common allergy in horses resembling atopic dermatitis and studies have shown that first exposure to allergens in adult life results in an increased incidence of IBH. The aim of the present study was to characterize circulating CD4^+^CD25^high^FoxP3^+^cells in foals, evaluate their suppressive capability and their in vitro induction compared to adult horses. 19 foals (age range, 1–5 months), their adult mothers and six one-year-old horses (yearlings) were included in the study. The proportion of FoxP3^+^ cells within the circulating CD4^+^CD25^high^ population was significantly higher in foals (47%) compared to their mothers (18%) and to yearlings (26%). Treg cells from foals also displayed a higher suppressive capability. Furthermore, CD4^+^CD25^high^ cells in foals could be induced *in vitro* from CD4^+^CD25^−^ cells in a significantly higher proportion compared to mares. These cells also displayed a significantly enhanced suppressive capability. In summary these findings support the notion that exposure of horses to allergens during maturation of the immune system assists the establishment of induced (i)Treg driven tolerance.

## Introduction

The immune system of mammals is subject to continuous changes during life, particularly during the postnatal and senescent phases of life. Exposure to a range of stimuli during maturation of the immune system seems to be required for its physiological development [1, 2]. Accordingly, epidemiologic studies suggest that the risk of allergy development originates in early childhood [3, 4]. While it is still a matter of debate whether a high exposure to allergens in early life has a protective or predisposing role on the development of allergic diseases [5–8], experimental models suggest that resistance to allergy is driven by environmental allergen exposure [9]. Equine insect bite hypersensitivity (IBH) is a naturally occurring large animal model of allergy in Europe but does not occur in Iceland due to the absence of biting insects belonging to the genus *Culicoides* that cause IBH. However, Icelandic horses exported as adults to mainland Europe, where *Culicoides spp*. are present, have a >50% incidence of developing IBH. In contrast, their progeny born in mainland Europe has a <10% incidence of developing IBH [10]. Interestingly, a recent study demonstrates that the prevalence of IBH in horses imported from Iceland is influenced by the age at import that is the first exposure to the allergen [11, 12]. Horses imported as weanlings at an age of 7 to 10 months do not develop IBH more frequently than Icelandic horses born in Europe. This indicates that early life exposure to the causative allergens is required to prevent the development of IBH.

The composition of the neonatal immune system is heavily biased towards an innate reaction with innate myeloid and lymphoid cells dominating [13]. The changes after birth are particularly dramatic and exposure to a range of stimuli, such as pathogens, leads to predominantly adaptive immune reactions over time. Treg cells play a key role in balancing immune responses to maintain tolerance for example against allergens [14]. A recent study in humans suggests that the relationship between immune-modulatory T cell subsets, allergic sensitization and eczema is developmentally regulated [4]. Due to the limitations in obtaining blood samples from babies, relatively little is still known about changes in regulatory immunity after birth, particularly the generation of inducible Treg (iTreg) cells, which require the encounter of antigen [1].

Similar to other species, T cell development and cytokine production in foals depends on age and type of antigen encountered [15–17]. Previous studies have shown that the response of PBMC from foals after stimulation with mitogens is characterized by a deficient ability to produce strong Th1 or Th2 immune responses until around 3 months of age [15–20]. However, in response to intra-bronchial challenge with virulent *Rhodococcus equi*, new-born foals were able to mount a similar IFN-γ response with a decreased IL-4 expression as compared to adult horses [21–23]. Furthermore, a robust IL-10 expression in foals was detected following stimulation with Lipopolysaccharide (LPS) [24]. This suggests that T-cell responses of healthy foals may be biased towards regulatory immune responses, which are important for inducing tolerance to self-antigens as well as environmental antigens such as *Culicoides*. We have recently demonstrated that IBH is associated with a reduced regulatory immune response specific for the causative allergens [25]. Moreover, Treg cells in IBH-affected horses displayed a strongly impaired ability to suppress *Culicoides*-stimulated proliferation of cells [26]. These findings together with the low incidence of IBH in Icelandic horses imported as foals, have led us to hypothesize that exposure to the allergens during maturation of the immune system is required for establishing tolerance against IBH. So far, however, no studies have been undertaken to analyse the presence and function of inducible Treg cells in foals or young animals in other livestock species. Here, we characterize circulating Treg cells in juvenile horses, their induction *in vitro* and determine their suppressive function compared to adult horses.

## Materials and Methods

### Study group

Blood samples from 44 clinically healthy horses were obtained by jugular venepuncture and collected into sterile sodium heparin containing vacuette tubes (Greiner Bio-One vacuette GmbH, St-Gallen, Switzerland) by a veterinarian. The horses belonged to various breeds (Icelandic, Swiss Warmblood and Franches-Montage horses) and were all born and living in Switzerland. The adult horses and yearlings were regularly dewormed and vaccinated. Subgroups of horses were randomly selected for individual parts of the study as described below. The study was approved by the animal experimentation ethics committee of the canton of Berne, Switzerland.

### Examination of regulatory T cells in freshly isolated and in stimulated PBMC

Peripheral blood mononuclear cells (PBMC) were isolated by Ficoll gradient centrifugation as described [27]. Horses included in this experiment were 14 foals (median age 3.5, range 1–5 months), 14 mares (median age 19, range 12–26 years) and 6 yearlings.

4x10^6^ freshly isolated PBMC were examined for the presence of circulating regulatory T cells. This was performed by measurement of the expression of CD4, CD25 and FoxP3 (S1 and S2 Figs.) by intracellular staining using flow cytometry as described [28].

4x10^6^ freshly isolated PBMC were cultured in the absence (mock) or presence of a combination (cocktail) of recombinant human IL-2 (rh.IL-2, Peprotech, London, UK, 100 U / ml), recombinant human TGF-β1 (rh.TGF-β1, Peprotech EC, 2ng / ml) and concanavalin A (conA, Sigma-Aldrich, St. Louis, MO, USA; 5 μg / ml). At day 4, cells were analysed for FoxP3 expression (S2 Fig.) by different subsets of CD4^+^ cells as described below.

All cell cultures were performed in RPMI 1640 containing 10% FCS, penicillin (100 IU/ml), streptomycin (100 μg/ml), nonessential amino acids (1%), MEM vitamins (100 μM), sodium pyruvate (1 mM) and 2-mercapoethanol (50 μM).

### Sorting experiments

Horses included in this experiment were 5 foals (3 females and 2 males; age range 1–2 months) and 5 mares (median age 15, range 5–17 years). PBMC were isolated as mentioned above. Enrichment of CD4^+^ cells was performed by positive selection using magnetic antibody cell separation (MACS), followed by indirect FITC labelling of CD4 and CD25 staining as described [28]. Using a BD FACSAria sorter, CD4^+^CD25^−^, CD4^+^CD25^dim^ and CD4^+^CD25^high^ lymphocytes were identified and sorted (S3 Fig.) as described previously [28].

### Measurement of T cell proliferation and FoxP3 and IL-10 expressions by flow cytometry

To study the suppressive function of Treg we used an allogeneic mixed leucocyte reaction (MLR) as described before [28]. In brief, allogeneic PBMC from another horse than any included in our study were used as stimulators of an allogeneic reaction. PBMC were therefore isolated and irradiated (44 Gy) using a Gammacell 40 research irradiator (Department of Clinical Research, University of Bern; Switzerland). 5x10^5^ sorted CD4^+^CD25^−^ responder cells were labelled with carboxyfluorescein diacetate succinimidyl ester (CFSE, 5μM, Sigma-Aldrich; http://www.sigmaaldrich.com). In a 24 well plate (Sarstedt, Nümbrecht, Germany), the labelled cells were cultured alone or with autologous CD4^+^CD25^dim^ or CD4^+^CD25^high^ putative suppressor cells in a ratio of 1: 0.25, in the presence of 5X10^5^ allogeneic PBMCs At day 4, the cells were harvested, stained for CD25 followed by intracellular staining for FoxP3 and IL-10 as described [28]. Cells were analysed using a LSRII flow cytometer (Becton-Dickinson) as described [28].

Data analysis was performed using Flowjo software (TreeStar Inc-Ashland). A first gate was set around CD4^+^CFSE^+^ cells to exclude the stimulator cells and define CD4^+^CD25^−^ cells. The percentage proliferation of CFSE-labelled CD4^+^CD25^−^ cells was determined (S4A Fig.). A second further gate was set around CD4^+^CFSE^−^CD25^high^ cells defining CD4^+^CD25^high^ cells. In case of expanded CD25^high^ cells, only one gate was required. The percentage of FoxP3^+^ or IL-10^+^ cells was determined within CD4^+^CD25^high^ cells (S4B Fig.). Accordingly, the percentage of **FoxP3** ^**+**^IL-10^−^, **IL-10** ^**+**^FoxP3^−^ **and FoxP3** ^**+**^ **IL-10** ^**+**^ expressing cells was determined and the percentage of these cells within the gated CD25^high^ cell population calculated.

### *In vitro* expansion and induction of CD4^+^CD25^high^ cells

Horses included in these experiments were the same as those used for the sorting experiments. For the i*n vitro* expansion of circulating CD4^+^CD25^high^ cells, these were sorted from freshly isolated CD4^+^ T cells as mentioned above. 2.5x10^5^ CD4^+^CD25^high^ cells were cultured in the absence (mock) or presence of the cocktail described above for four days. Cell numbers were counted by haemocytometer before and after culture to determine the expansion of these cells. The expanded CD4^+^CD25^high^ cells were stained for CD25, FoxP3 and IL-10 and the percentage of positive cells was measured by flow cytometry as described above. A representative flow cytometry example is shown in S5 Fig.

For the *in vitro* induction of CD4^+^CD25^high^ cells from CD4^+^CD25^−^ cells, freshly isolated CD4^+^ T cells were sorted into CD4^+^CD25^−^ as mentioned above and 1x10^7^ CD4^+^CD25^−^ cells were cultured with the cocktail. After four days, the cultured cells were stained for CD25 and re-sorted. The proportion of induced (_I_) CD4^+^CD25^high^ cells was analysed. The ability of _I_CD4^+^CD25^high^ cells to suppress proliferation of freshly isolated CFSE-labelled CD4^+^CD25^−^ T cells was determined in an MLR assay as described above. In addition, the expression of FoxP3 and IL-10 by _I_CD4^+^CD25^high^ cells was determined by flow cytometry (S6 Fig.) as described above.

### Statistical analysis

NCSS 2007 software program (NCSS, Kaysville, Utah 84037, USA) was used for statistical analyses. Descriptive statistics by group were run on the data and Kolmogrov–Smirnov test showed that the data are not normally distributed. Therefore, non-parametric tests were chosen. On each occasion, p values ≤ 0.05 were regarded as significant.

**Fig 1:**
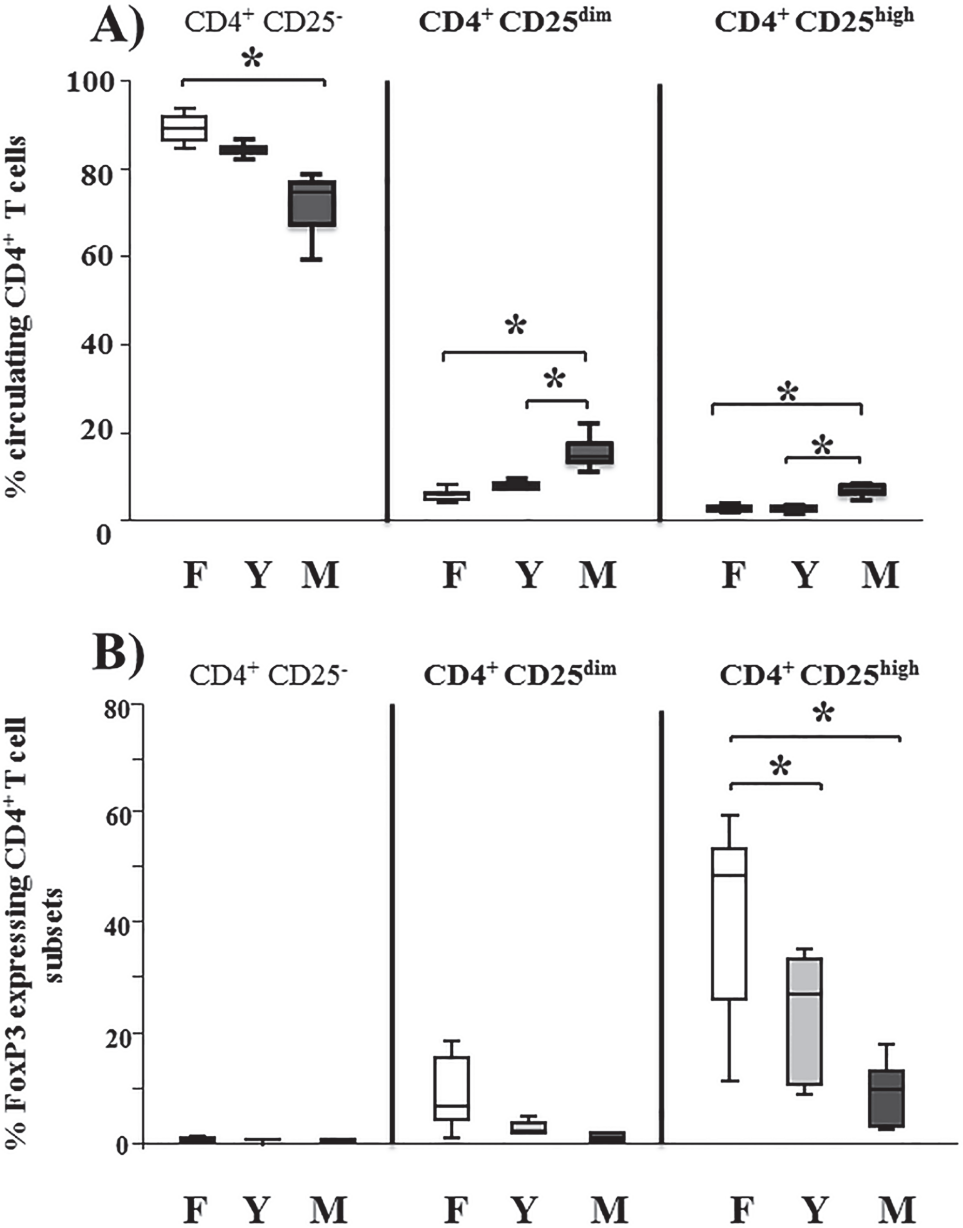
Proportion of circulating CD4+ T cell subpopulations and expression of FoxP3 in freshly isolated PBMC. 4x10^6^ freshly isolated peripheral blood mononuclear cells (PBMC) from foals (**F**, n = 14, age 1–5 months), yearlings (**Y**, n = 6) and mares (**M**, n = 14, age 12–26 years) were stained for CD4, CD25 and FoxP3. The proportion of *CD4* ^*+*^ *CD25* ^*−*^, *CD4* ^*+*^ *CD25* ^*dim*^ and *CD4* ^*+*^ *CD25* ^*high*^ T cells **(A)** and expression of FoxP3 **(B)** within these 3 subpopulations were determined using flow cytometry. While differences between foals and yearlings do not surmount to more than a trend, it became obvious that there is a significant shift in T cell populations between foals and adult animals across all subpopulations. While mares have around twice the amount of CD4^+^CD25^high^ cells in the blood, foals have around twice the amount of FoxP3^+^ cells, indicating a shift from nTreg towards activated T cells within this compartment. Results from all horses examined are presented as box plots, whereby, the centre horizontal line of the box plot marks the median of the sample. The edges of the box mark the first and third quartiles and the whiskers define the upper adjacent value which is the largest observation that is less than or equal to the 75^th^ percentile plus 1.5 times the interquartile range (IQR) and the lower adjacent value which is the smallest observation that is greater than or equal to the 25^th^ percentile minus 1.5 times IQR. Comparisons were performed using Kruskal-Wallis Multiple-Comparison Z value Test with Bonferroni correction. The asterisk indicates statistical significance differences of p ≤ 0.05.

**Fig 2:**
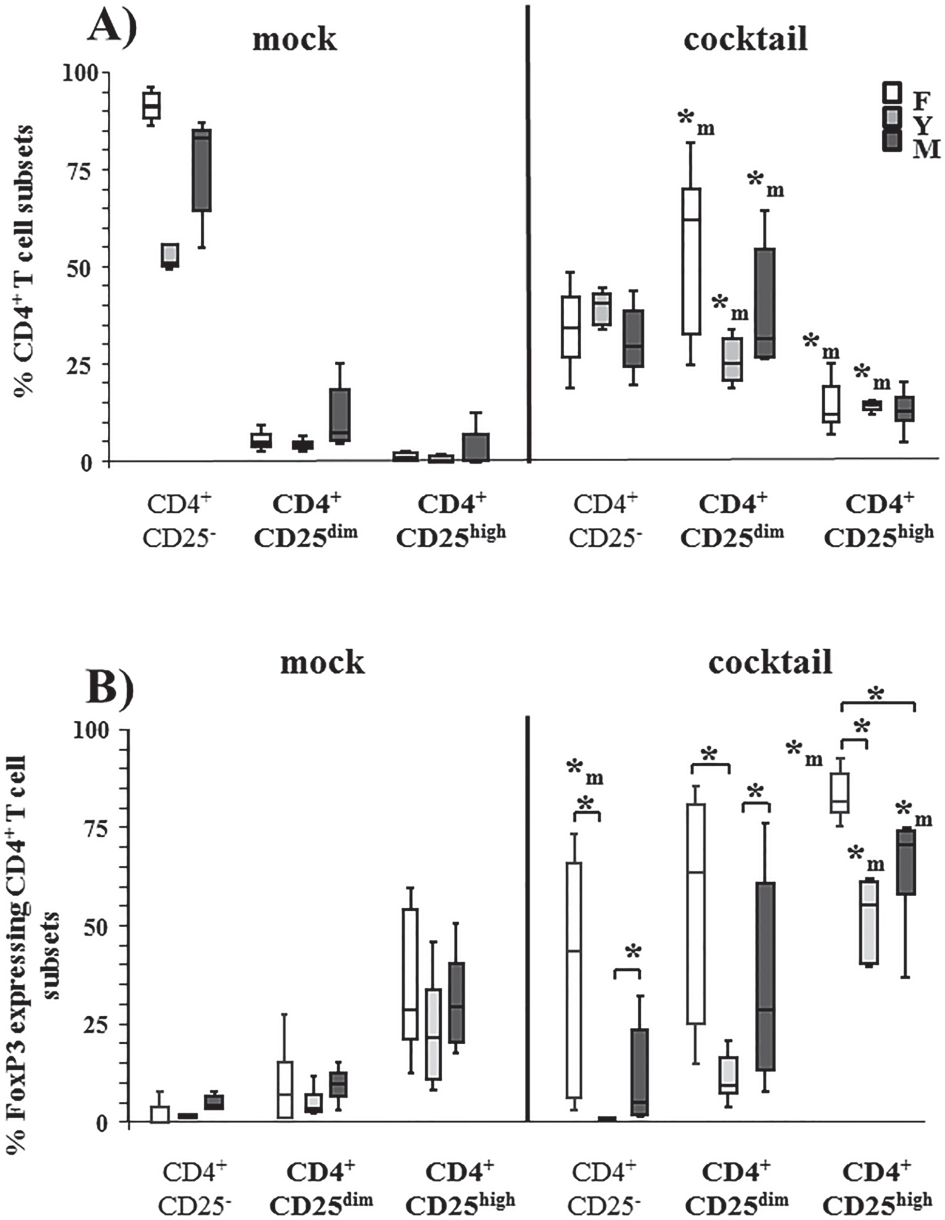
Proportion of CD4+ T cell subpopulations and expression of FoxP3 within these subpopulations following stimulation. 4x10^6^ freshly isolated PBMC from foals **(F)**, yearlings **(Y)** and from mares **(M)** were left either un-stimulated (mock) or stimulated with a combination of conA, r.IL-2 and r.TGF-β1 (cocktail) for four days. Horses included are the same as mentioned in **Fig. 1**. Proportion of *CD4* ^*+*^ *CD25* ^*−*^, *CD4* ^*+*^ *CD25* ^*dim*^ and *CD4* ^*+*^ *CD25* ^*high*^ T cells **(A)** and expression of FoxP3 **(B)** within these subpopulations were determined and presented as in **Fig. 1**. In addition, (*m) indicates a significant difference to mock within the respective cell subpopulation of the same horse group using non-parametric Wilcoxon signed-rank paired t-test.

**Fig 3:**
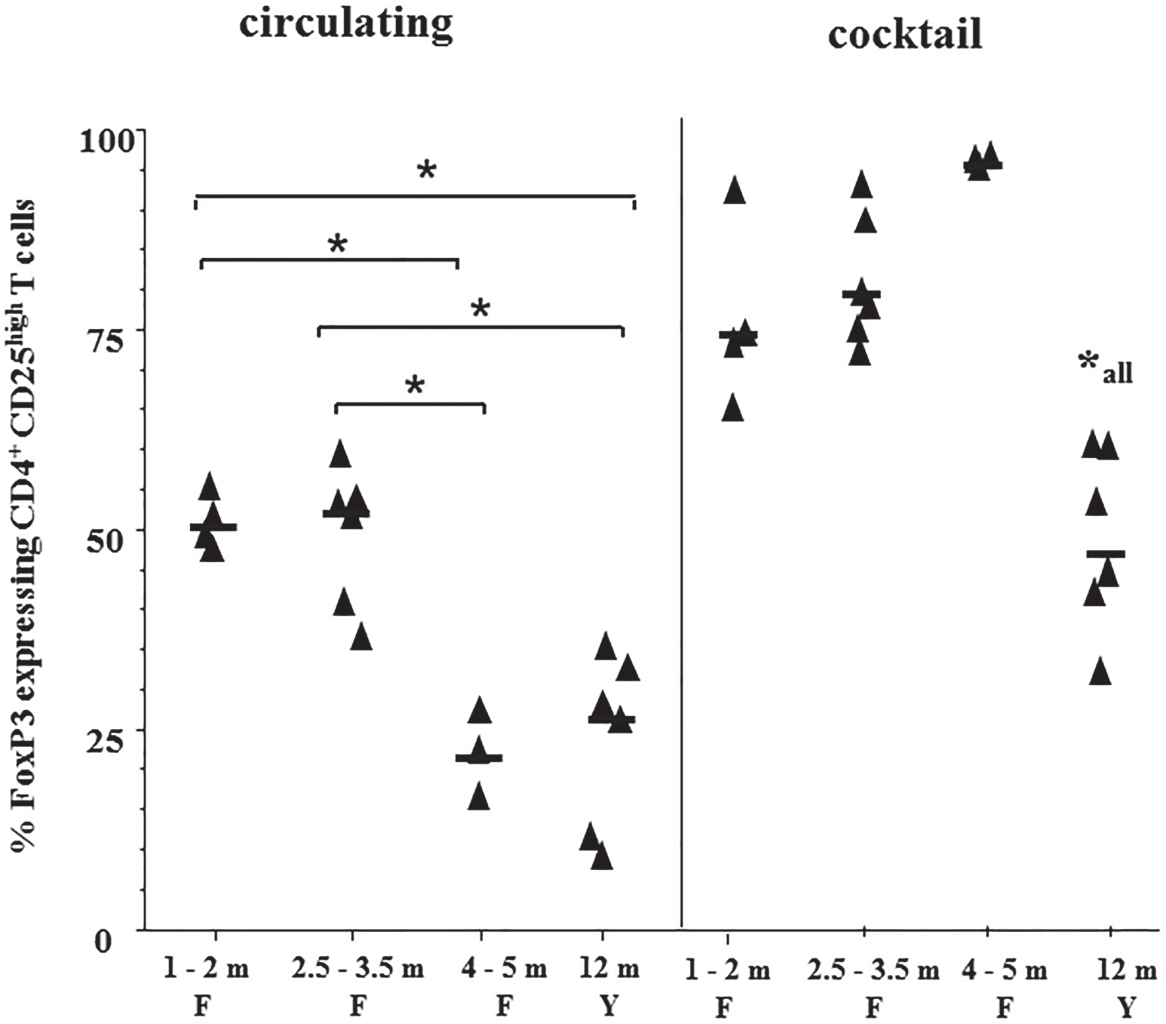
Influence of the age of foals on the proportion of CD4+CD25high FoxP3+ cells. Results from foals and yearlings reported in **Figs. 1 and 2** were grouped according to their age into four categories: Foals, 1–2 months (F), 2.5–3.5 months, 4–5 months and yearlings 12 months (Y). The effect of the age group on the percentage of **CD4** ^**+**^ **CD25** ^**high**^ **FoxP3** ^**+**^ cells (circulating) in freshly isolated as well as cocktail stimulated (cocktail) PBMC was examined using one way ANOVA. Comparisons between the four groups were performed using Kruskal-Wallis Multiple-Comparison Z value Test with Bonferroni correction. Each data point represents 1 horse, horizontal bars within values denote medians. An asterisk with line indicates statistical significance difference between the groups of horses. *all indicates significant difference of yearlings to the 3 groups of foals. *p ≤0.05 was considered significant.

The non-parametric ANOVA Kruskal-Wallis multiple-Comparison Z-Value Test was used to compare the differences in the frequency of the three CD4^+^ T cell subsets (CD4^+^CD25^−^, CD4^+^CD25^dim^, CD4^+^CD25^high^) **(Fig. 1A)** and the expression of FoxP3 by different CD4^+^ T cell subsets **(Figs. 1B and 2B)**. This test was also used to compare the expression of FoxP3 by the different age groups of foals **(Fig. 3B)**. The Bonferroni test was used to correct for multiple comparisons.

**Fig 4:**
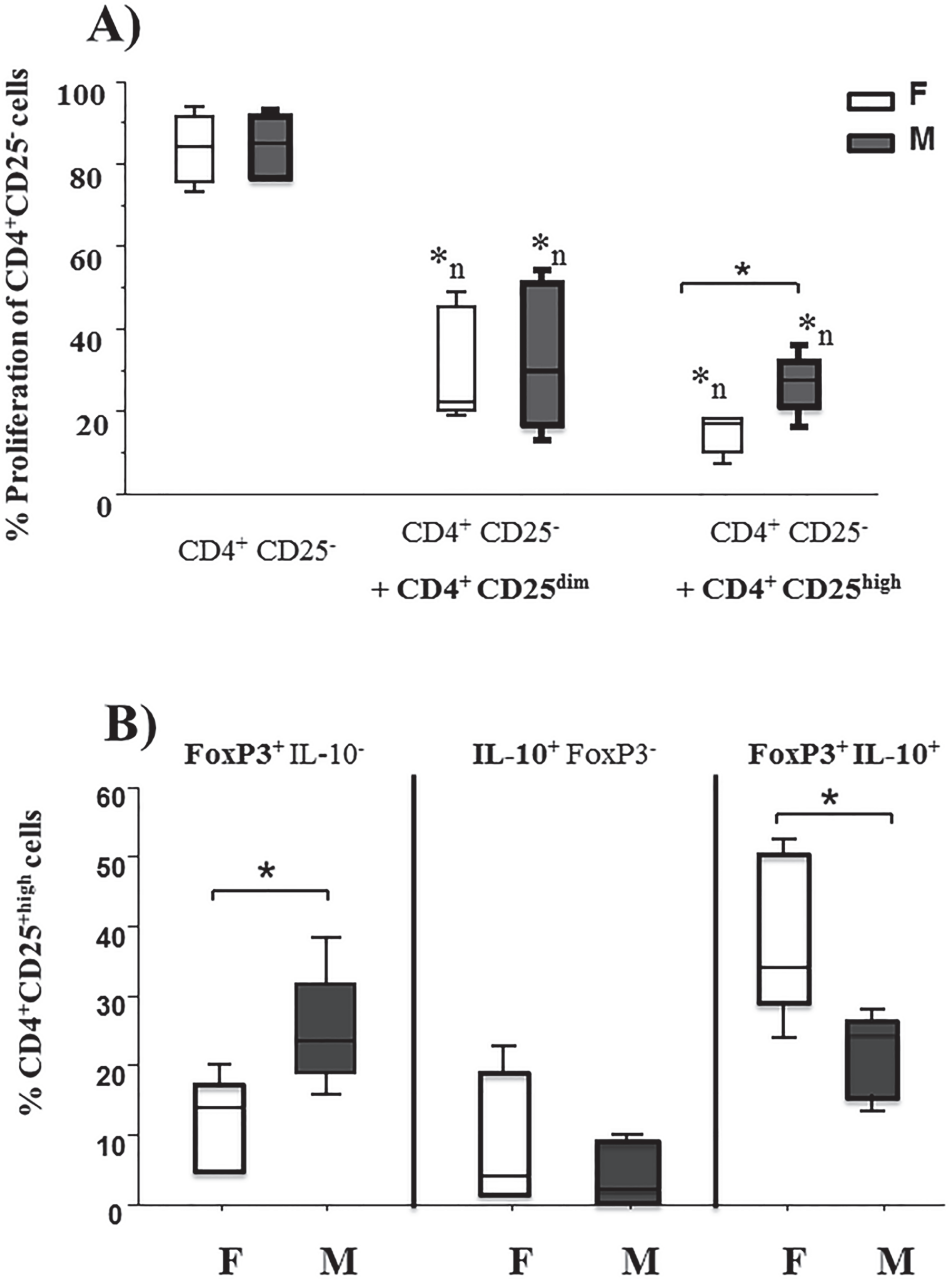
Suppressive ability of CD4^+^CD25^+^ cells and proportion of IL-10^+^FoxP3^+^ within CD4^+^CD25^high^ cells from foals and mares. 5x10^5^ CD4^+^CD25^−^ lymphocytes sorted from freshly isolated PBMC of foals (**F**, n = 5, age 1–2 months) and mares (**M**, n = 5, 5–17 years) were labelled with carboxyfluorescein diacetate succinimidyl ester (CFSE) and cultured with 5x10^5^ irradiated allogeneic PBMC alone **(CD4** ^**+**^ **CD25** ^**−**^) or in the presence of CD4^+^CD25^dim^ **(CD4** ^**+**^ **CD25** ^**−**^ **+ CD4** ^**+**^ **CD25** ^**dim**^) or CD4^+^CD25^high^ **(CD4** ^**+**^ **CD25** ^**−**^ **+ CD4** ^**+**^ **CD25** ^**high**^) cells in a ratio of 1: 0.25. After 4 days, the cells were harvested and stained for FoxP3 and IL-10. The percentage of proliferation **(A)** and of single positive **FoxP3** ^**+**^IL-10^−^, single positive **IL-10** ^**+**^FoxP3^−^ and double positive **IL-10** ^**+**^ **FoxP3** ^**+**^ **(B)** was measured by flow cytometry. Results are shown as percentage. Results from all horses examined are presented as box plots. An asterisk with line indicates statistical significance difference. *n indicates significance difference to **CD4** ^**+**^ **CD25** ^**−**^ cells within same group of horses. Comparisons were performed using non-parametric Mann-Witney U test. *p ≤0.05 was considered significant.

**Fig 5:**
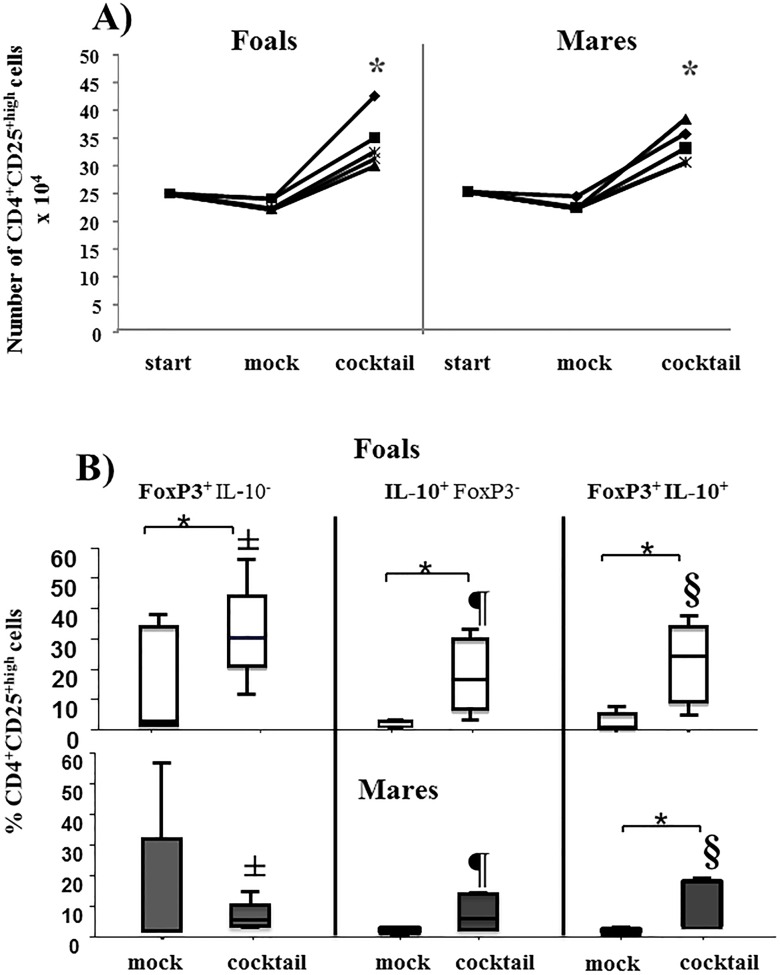
Expansion of circulating CD4^+^CD25^high^ cells from foals and mares. 2.5x10^5^ CD4^+^CD25^high^ lymphocytes sorted from freshly isolated PBMC of foals (**F**, n = 5, age 1–2 months) and mares (**M**, n = 5, age 5–17 years) were left either un-stimulated (mock) or stimulated with cocktail. **A)** The cells were counted before **(start)** and after culture **(mock, cocktail)** using a haemocytometer and Trypan blue to exclude dead cells. Results are shown as number of cells, dots represent individual horses. * Indicates significant differences to mock and start using paired t-test. **B)** After 4 days, the cells were harvested and stained for FoxP3 and IL-10. The percentage of single positive **FoxP3** ^**+**^IL-10^−^, single positive **IL-10** ^**+**^FoxP3^−^ and double positive **IL-10** ^**+**^ **FoxP3** ^**+**^ was measured by flow cytometry. Results are shown as percentages and presented as box plots. An asterisk with line indicates significant difference of cocktail to mock within the respective cell subpopulation and same horse group using non-parametric Wilcoxon signed-rank paired t-test. ±, ¶, § indicate statistical significance difference between foals and mares within the respective cell population using non-parametric Mann-Witney U test. *p ≤0.05 was considered significant.

**Fig 6:**
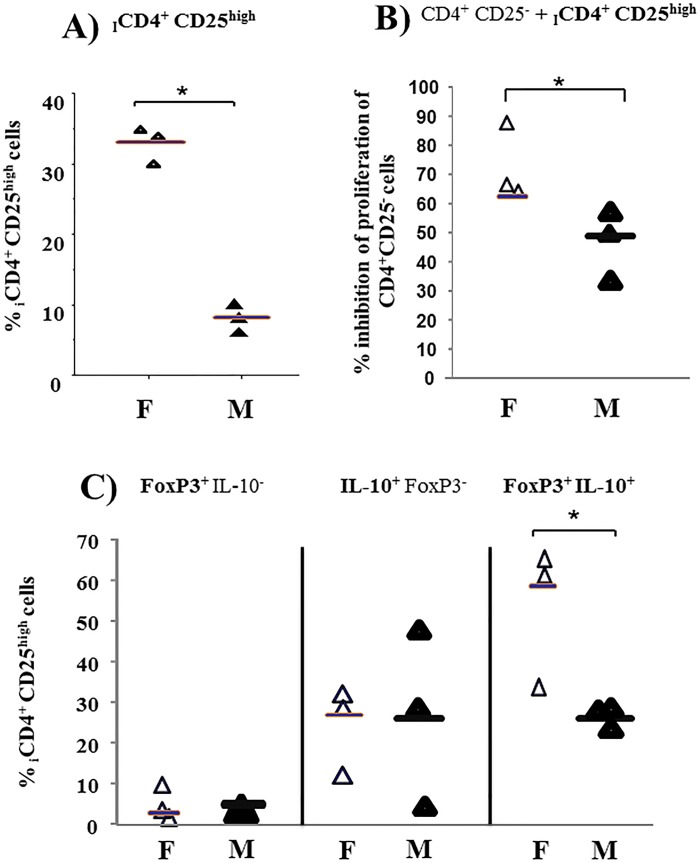
Induction of CD4^+^CD25^high^ cells from CD4^+^CD25^−^ cells from foals and mares. 1x10^7^ CD4^+^CD25^−^ lymphocytes sorted from freshly isolated PBMC of foals (**F**, n = 3, age 1–2 months) and mares (**M**, n = 3, 5–17 years) were cultured with the cocktail. 4 days later, the cells were harvested, stained for CD25 and resorted for induced CD4^**+**^CD25^high^ (_**I**_ **CD4** ^**+**^ **CD25** ^**high**^) cells. **A)** Proportion of _I_CD4^+^CD25^high^ cells from foals and mares were measured using flow cytometry. 5x10^5^ CD4^**+**^CD25^**−**^ cells sorted from freshly isolated PBMC were labelled with CFSE and cultured with 5x10^5^ irradiated allogeneic PBMC alone **(CD4** ^**+**^ **CD25** ^**−**^) or in the presence of _I_CD4^**+**^CD25^high^ **(CD4** ^**+**^ **CD25** ^**−**^ **+**_**I**_ **CD4** ^**+**^ **CD25** ^**high**^) cells in a ratio of 1: 0.25. After 4 days, the cells were harvested and stained for FoxP3 and IL-10. **B) P**ercentage of proliferation was measured by flow cytometry. Results are shown as percentage inhibition of proliferation of **CD4** ^**+**^ **CD25** ^**−**^ cells by _I_CD4^**+**^CD25^high^ cells following stimulation with irradiated allogeneic PBMC. **C)** Percentage of single positive **FoxP3** ^**+**^IL-10^−^, single positive **IL-10** ^**+**^FoxP3^−^ and double positive **IL-10** ^**+**^ **FoxP3** ^**+**^ was measured by flow cytometry. Each data point represents 1 horse horizontal bars within values denote medians. An asterisk with line indicates statistical significance difference. Comparisons were performed using non-parametric Mann-Witney U test. *p ≤0.05 was considered significant.

The non-parametric Mann-Witney U test was used to compare the differences in the percentage of proliferation **(Fig. 4A)**, frequency of CD4^+^CD25^high^ cells expressing-FoxP3, IL-10 **(Figs. 4B, 5B and 6C),** proportion of induced CD4^+^CD25^high^ cells **(Fig. 6A)** and percentage of inhibition by induced CD4^+^CD25^high^ cells ***(*Fig. 6B)** between foals and mares. The non-parametric Wilcoxon signed-rank paired t-test was used to compare the differences in the frequency of CD4^+^CD25^−^, CD4^+^CD25^dim^, CD4^+^CD25^high^ cells **(Fig. 2A)**, the expression of FoxP3 by different CD4^+^ T cell subsets **(Fig. 2B)**, number of expanded CD4^+^CD25^high^ cells **(Fig. 5A)** and frequency of CD4^+^CD25^high^ cells expressing-FoxP3, IL-10 **(Fig. 5B)** between mock and cocktail within the same horse group.

## Results

### Circulating CD4^+^CD25^high^ T cells contain a significantly higher proportion of FoxP3^+^ cells in foals compared to adult horses

We had shown previously the presence of circulating CD4^+^CD25^high^FoxP3^+^ T cells in adult horses [28]. However, studies on the development of these cells in foals were still lacking. Therefore, we first examined the existence of circulating CD4^+^CD25^high^FoxP3^+^ T cells in foals (age range, 3–12 weeks) and compared it with that from their dams and yearlings. **Fig. 1A** shows the presence of CD4^+^CD25^dim^ and CD4^+^CD25^high^ cells in freshly isolated PBMC from foals and yearlings as measured by flow cytometry. Like in mares, the CD4^+^CD25^−^ cells from foals and yearlings represent the major subpopulation of T cells. However, the proportion of circulating CD4^+^CD25^dim^ and CD4^+^CD25^high^ cells was significantly higher in mares (median, range; 6, 4–8%) compared to foals (2, 1–4%) and yearlings (3, 2–4%). Conversely, there was a significantly higher proportion of FoxP3^+^ cells within CD4^+^CD25^high^ subpopulation from foals (47, 10–59%) compared to yearlings (26, 8–35%) and to mares (10, 4–18%) **(Fig. 1B)**, indicating a higher proportion of nTreg cells in this compartment. No significant differences were detected in the number of FoxP3^+^ cells within CD4^+^CD25^dim^ or CD4^+^CD25^−^ between the 3 groups of horses, although there was a trend for an increased FoxP3 expression in CD4^+^CD25^dim^ cells of foals, indicative for the presence of iTreg.

### Ability to up-regulate FoxP3^+^ T cells in vitro is higher in foals compared to adult horses

We had shown previously in adult horses that FoxP3 expression could be induced *in vitro* with a combination of r.IL-2, r.TGF-β1 and conA (cocktail) [28]. Accordingly, it was of interest to compare the ability to modulate the expression of FoxP3 within PBMC from foals. Therefore, we have examined the proportion of CD4^+^ cell subpopulations as well as the FoxP3^+^ expression within CD4^+^CD25^+^ and CD4^+^CD25^−^ populations after *in vitro* stimulation of PBMC with the cocktail. **Fig. 2A** shows a significant increase in the proportion of CD4^+^CD25^dim^ cells from foals, yearlings and mares upon stimulation with the cocktail compared to un-stimulated cells (mock). Interestingly, the proportion of CD4^+^CD25^high^ cells was significantly increased by the cocktail stimulation in foals and yearlings but not in mares, the latter confirming previous results in adult horses [20]. Upon *in vitro* culture, significant differences in the proportion of CD4^+^CD25^−^, CD4^+^CD25^dim^ and ^high^ cells were no longer detected between the 3 groups of horses **(Fig. 2A)** compared to freshly isolated cells **(Fig. 1A)**. Furthermore, stimulation with the cocktail significantly up-regulated the number of FoxP3-expressing cells in the CD4^+^CD25^−^ and CD4^+^CD25^high^ populations from foals **(Fig. 2B)**. In contrast to foals, a significant up-regulation of FoxP3 in yearlings and mares was only found within CD4^+^CD25^high^ cells. Notably, upon stimulation with the cocktail the proportion of FoxP3-expressing CD4^+^CD25^high^ cells was significantly higher in foals than in yearlings and mares. Here, the number of FoxP3-expressing cells by CD4^+^CD25^high^ cells reached up to 90%.

### Significant influence of the age of foals on the expression of FoxP3

We have further examined the influence of age on the expression of FoxP3 within CD4^+^CD25^high^ cells in foals during the first 6 months of life. The foals used in the previously mentioned experiments were grouped into 3 categories: 1–2 months, 2.5–3.5 months and 4–5 months and compared with yearlings. There was a significant influence of age on the proportion of FoxP3^+^cells within CD4^+^CD25^high^ in freshly isolated (circulating, P ≤ 0.0001) as well as stimulated (cocktail, P ≤ 0.002) PBMC.

**Fig. 3** shows a significantly higher proportion of circulating CD4^+^CD25^high^ FoxP3^+^ in the age groups of 1–2 months and 2.5–3.5 months compared to the age group 4–5 months and yearlings. No significant difference was detected between the age group 4–5 months and yearlings, indicative for a rapid adaptation in the first 6 rather than 12 months. While, there were no significant differences in the proportions of CD4^+^CD25^high^ FoxP3^+^ cells between the 3 age groups of foals upon PBMC stimulation with the cocktail, there was a significant difference between them and the yearlings.

### CD4^+^CD25^high^ cells from foals possess an enhanced suppressive capability and express more IL-10

Since have shown that circulating CD4^+^CD25^high^ cells from foals between 1 and 2.5 months of age contain the highest proportion of FoxP3, it was of interest to compare the capability of these cells to suppress the proliferation of CD4^+^CD25^−^ T cells with CD4^+^CD25^high^ cells from mares. **Fig. 4A** displays the results of allogeneic MLRs without or with the addition of sorted CD4^+^CD25^dim^ or CD4^+^CD25^high^ cells. In foals and mares, the addition of autologous CD4^+^CD25^dim^ or CD4^+^CD25^high^ cells significantly decreased the proliferation of CD4^+^CD25^−^ cells. More so, CD4^+^CD25^high^ cells from foals showed a significantly higher ability (18, 8–20% proliferation) for suppression compared to mares (29, 18–38%).

This finding was further investigated by determining the proportion of **FoxP3** ^**+**^IL-10, **IL-10** ^**+**^FoxP3^−^ and **FoxP3** ^**+**^ **IL-10** ^**+**^—expressing cells in this compartment after co-cultivation with allogeneic PBMC. Interestingly, compared to mares foals show a significantly higher proportion of IL-10 expressing cells within CD4^+^CD25^high^FoxP3^+^ cells **(Fig. 4B)**. In contrast, CD4^+^CD25^high^ cells from mares showed a significantly higher proportion of **FoxP3** ^**+**^ **IL-10** ^**−**^ cells than foals. No significant differences were detected in the proportion of any of these cell populations within CD4^+^CD25^dim^ cells between foals and mares (data not shown).

### *In vitro* expansion of foal CD4^+^CD25^high^ cells is associated with an increase in FoxP3^+^ and IL-10^+^ cells.

Based on previous studies (17, 20), we have examined the ability to expand circulating CD4^+^CD25^high^ cells *in vitro*. First, the CD4^+^CD25^high^ cells were counted before and after culture to verify their expansion **(Fig. 5A)**. While indeed, stimulation with a cocktail of conA, IL-2 and TGF-β1 resulted in a significant increase (2-fold) in the number of CD4^+^CD25^high^ cells, the increase was similar between foals and mares.

Next, the proportion of **FoxP3** ^**+**^IL-10^−^, **IL-10** ^**+**^FoxP3^−^ and **IL-10** ^**+**^ **FoxP3** ^**+**^-expressing CD4^+^CD25^high^ cells was compared between foals and mares **(Fig. 5B)**. Upon stimulation, there was a significant increase in the amount of **FoxP3** ^**+**^IL-10^−^, **IL10** ^**+**^FoxP3^−^ and **FoxP3** ^**+**^ **IL-10** ^**+**—^expressing CD4^+^CD25^high^ cells from foals compared to mock. However, in mares there was a significant increase only in the proportion of **FoxP3** ^**+**^ **IL-10** ^**+**-^expressing CD4^+^CD25^high^ cells. Moreover, the proportion of **FoxP3** ^**+**^IL-10^−^, **IL-10** ^**+**^FoxP3^−^ and **FoxP3** ^**+**^ **IL-10** ^**+**^ cells within expanded CD4^+^CD25^high^ cells was significantly higher in foals compared to mares.

### CD4^+^CD25^−^ cells from foals are skewed to become FoxP3^+^IL-10^+^ suppressive cells upon stimulation

We have also examined the ability to induce CD4^+^CD25^high^ Treg from CD4^+^CD25^−^ cells in foals and mares. CD4^+^CD25^−^ cells sorted from freshly isolated PBMC from foals or mares were cultured *in vitro* with cocktail for 4 days. This resulted in the induction of a significantly higher proportion of _I_CD4^+^CD25^high^ cells (median, range; 34, 10–40%) in foals compared to mares (_I_CD4^+^CD25^high^, 8, 5–13%) **(Fig. 6A)**. Furthermore, **Fig. 6B** shows a significantly increased ability of _I_CD4^+^CD25^high^ (67, 64–87%) cells from foals to suppress proliferation of CD4^+^CD25^−^ cells in a MLR. Similar to the expansion, _I_CD4^+^CD25^high^ cells from foals contain a significantly higher proportion of **FoxP3** ^**+**^ **IL-10** ^**+**^ cells compared to mares **(Fig. 6C)**.

## Discussion

In 1953, Billingham and Medawar showed that mice injected with allogeneic splenocytes at birth were subsequently able to accept skin allografts from the same donor strain [29]. Moreover, dizygotic cattle twins accepted skin grafts from each other [30]. This led to the belief that the neonatal period represents an ontogenic window for tolerance induction towards self- and foreign antigens [31]. In mice the immediate post-natal removal of the thymus results in a failure to induce tolerance, which can be restored through adoptive transfer of T cells [32]. In humans, the foetal generation of Treg cells is believed to play an important role in suppressing T cell responses during development, establishing tolerance [33, 34]. Due to the difficulties in obtaining samples from healthy subjects, relatively little is known about changes in the regulatory immunity in humans after birth and most studies compare cord blood cells with adult Treg cells. It has been suggested in these studies that nTreg cells from cord blood have a reduced suppressive capacity [35–37], but it needs to be taken into account that CD4^+^CD25^+^ cord blood cells proliferate in response to anti-CD3/CD28 mAb-coated beads [36], which is not a common feature of Treg cells.

In the present study, we have examined circulating Treg cells in the large animal model of horses prior to adolescence for the first time. While the significantly lower proportion of circulating CD4^+^CD25^high^ cells in foals reflects their lack of encountered foreign and dangerous antigens, 47% of these cells were FoxP3^+^ compared to only 10% in mares **(Fig. 1)**. This is in accordance with our previous data, demonstrating that CD4^+^CD25^high^ cells in adult horses are heterogeneous containing only a minority of FoxP3^+^ cells [28]. While we have not studied the immediate post-natal period, we can assume that the circulating FoxP3^+^ cells in foals *in vivo* resemble the default generation of neonatal regulatory T cells described for mice *in vitro* [31]. Accordingly, the default phenotype of circulating CD4^+^CD25^high^ cells in neonatal foals resembles Treg cells rather than activated T cells.

Similar to adult animals, the combination of IL-2, TGF-β1 and conA (cocktail) induced a significantly higher proportion of FoxP3 within CD4^+^CD25^high^ cells **(Fig. 2)**. More so, the proportion of CD4^+^CD25^high^ cells was also significantly up-regulated reaching a similar frequency as in mares. Not unexpectedly the expression of FoxP3 in CD4^+^CD25^+^ cells changed inversely proportional to the age of foals in that the proportion of circulating Treg cells dropped significantly by the age of 4–5 months **(Fig. 3)**. However, there were no significant differences between the age groups of foals, in the proportion of iTreg cells induced upon stimulation with the cocktail, indicating an increased ability of foals to up-regulate FoxP3 expression compared to yearlings or adult animals. While this suggests a change from nTreg towards iTreg in the composition of the regulatory compartment, the immune system of young horses remains skewed towards tolerance induction.

CD4^+^CD25^high^ cells from the earliest (1–2 months age) foal group exhibit a stronger suppressive capability than those from adult horses **(Fig. 4)**. This was associated with a higher frequency of **FoxP3** ^**+**^ **IL-10** ^**+**^ cells, which is in accordance with our previous results that the frequency of **FoxP3** ^**+**^ **IL-10** ^**+**^ cells determines the suppressive ability of CD4^+^CD25^high^ cells in horses [26]. This is also in agreement with an earlier study that detected an increase number of IL-10^+^ cells in foals [24] and indicates a rapid activation of the Treg compartment after birth.

Similar to previous results using adult CD4^+^CD25^high^ cells we [28], could demonstrate that these cells from foals can be expanded *in vitro* **(Fig. 5)**. The degree of expansion was consistent with studies in other species [38–40].

Importantly, CD4^+^CD25^high^ Treg cells could also be induced from CD4^+^CD25^−^ cells in significantly higher numbers in foals upon *in vitro* stimulation by cocktail. This reflects data in mice, which showed that the frequency of induced CD4^+^CD25^+^ cells was increased 6-fold in neonates [31]. More so, _I_CD4^+^CD2^high^ cells from foals were more suppressive **(Fig. 6)**, which further underlines that CD4^+^CD25^−^ cells from foals are initialised to become Treg cells upon stimulation.

Taken together, the results demonstrate the presence of an increased proportion of functionally mature FoxP3^+^ cells within the circulating CD4^+^CD25^high^ Treg cells in foals during the first three months of life. The proportion of these FoxP3^+^ cells diminishes significantly at the age of 1 year. The circulating CD4^+^CD25^high^ Treg from foals exhibit a strong suppressive capacity, which can probably inhibit other cells like Th1 and Th2. This might explain the results of other studies [15–20], which have shown a deficient ability of foals to produce Th1 and Th2 cytokines until age of 3 months. Strikingly, CD4^+^CD25^−^ T cells from foals are biased to become CD4^+^CD25^high^ iTreg cells, suggesting that this might be the standard reaction of neonatal T cells in response to environmental foreign and self-antigens during the post-natal development of the immune system. Our study suggests that horses represent a suitable model to study of regulatory immunity in neonates. Future studies, using Icelandic horses as model population can investigate whether the default generation of Treg cells early in life is involved in establishing tolerance to *Culicoides* allergens.

## Supporting Information

S1 Fig Gating strategy of CD4^+^ CD25^+^ T cell subpopulations. Freshly isolated peripheral blood mononuclear cells (PBMC) from foals, yearlings and mares were stained for CD4 and CD25. The relative amount of CD4^+^CD25^−^ (N) CD4^+^CD25^dim^ (dim) and CD4^+^CD25^high^ (high) T cells was determined using flow cytometry as described before [28]. A gate was set around lymphocytes and gated cells were analysed for CD4 expression where another gate was positioned. CD4^+^ cells were subsequently analysed for CD25 expression. CD25^−^ cells were first distinguished from those with a dim fluorescence signal (dim) and additionally from those with a distinct (>10 fold) brighter fluorescence signal (high). Subsequently, three gates with small gaps between them were placed, defining the three CD4^+^ subpopulations.
(TIF)

S2 Fig Expression of FoxP3 within CD4^+^CD25^high^ cells. PBMC freshly isolated (0 h) or cultured for 4 days without (mock) or with the cocktail (cocktail) were stained for CD4, CD25 and FoxP3. The expression of FoxP3 within CD4^+^CD25^high^ cells is presented. Horses included are foals, yearlings and mares.
(TIF)

S3 Fig Purity of the sorted CD4^+^ subpopulations. After isolation of PBMC, CD4^+^ cells were enriched by positive magnetic cell separation (MACS). The CD4^+^ cells were stained for CD25 as described [28]. CD4^+^CD25^−^, CD4^+^CD25^dim^ and CD4^+^CD25^high^ T cells were identified and sorted as previously [28], using a more stringent gating than for phenotyping **(S1 Fig.)** to avoid cross contamination. Analysis of the three subpopulations after sorting, demonstrated > 98% purity for each of the three subpopulations.
(TIF)

S4 Fig Proliferation of CD4^+^CD25^−^ cells cultured in the presence and absence of CD4^+^CD25^high^ cells (A) and proportion of IL-10^+^ and FoxP3^+^ within CD4^+^CD25^high^ cells (B) from foals and mares. CD4^+^CD25^−^ lymphocytes sorted from freshly isolated PBMC of foals and mares as described in S3 Fig. were labelled with carboxyfluorescein diacetate succinimidyl ester (CFSE) and cultured without **(CD4** ^**+**^ **CD25** ^**−**^ **control,** top row) or with irradiated allogenic PBMC alone **(CD4** ^**+**^ **CD25** ^**−**^, middle row) or in the presence of sorted CD4^+^CD25^high^ **(CD4** ^**+**^ **CD25** ^**−**^ **+ CD4** ^**+**^ **CD25** ^**high**^, bottom row) cells. After 4 days, the cells were harvested and stained for FoxP3 and IL-10 or the relevant isotype controls. Analysis was performed using Flowjo software. **A)** The gated CFSE-labelled CD4^+^CD25^−^ cells were analysed for percentage proliferation by setting gates for proliferating **(FITC-A, APC-A Subset)** and non-proliferating **(FITC-A, APC-A Subset-1)** cells. **B)** The percentages of single positive **IL-10** ^**+**^FoxP3^−^ **(Q1)**, double positive **IL-10** ^**+**^ **FoxP3** ^**+**^ **(Q2)** and single positive **FoxP3** ^**+**^IL-10^−^ **(Q3)** within CD4^+^CD25^high^ cells were measured by flow cytometry.
(TIF)

S5 Fig Expression of FoxP3 and IL-10 within expanded CD4^+^CD25^high^ cells. CD4^+^CD25^high^ lymphocytes sorted from freshly isolated PBMC of foals and mares were left either un-stimulated (mock) or stimulated with cocktail. After 4 days, the cells were harvested and stained for FoxP3 and IL-10. The percentages of single positive **IL-10** ^**+**^FoxP3^−^ **(Q1)**, double positive **IL-10** ^**+**^ **FoxP3** ^**+**^ **(Q2)** and single positive **FoxP3** ^**+**^IL-10^−^ **(Q3)** were measured by flow cytometry.
(TIF)

S6 Fig Expression of FoxP3 and IL-10 within induced CD4^+^CD25^high^ cells. CD4^+^CD25^−^ lymphocytes sorted from freshly isolated PBMC of foals and mares were cultured with the cocktail for four days, harvested, stained for CD25 and resorted for induced CD4^**+**^CD25^high^ (_**I**_ **CD4** ^**+**^ **CD25** ^**high**^) cells. The _**I**_ **CD4** ^**+**^ **CD25** ^**high**^ cells were stained for FoxP3 and IL-10. The percentages of single positive **IL-10** ^**+**^FoxP3^−^ **(Q1)**, double positive **IL-10** ^**+**^ **FoxP3** ^**+**^ **(Q2)** and single positive **FoxP3** ^**+**^IL-10^−^ **(Q3)** were measured.
(TIF)

## References

[1] Prescott SL, Seroogy C. Ontogeny of immune development and its relationship to allergic diseases and asthma In: Adkinson NF J, Bochner BS, Burks AW, Busse WW, Holgate ST, Lemanske R.F. Jr., O'Hehir R.E., editors. Middleton's Allergy: Principles and Practice, Elseviers Inc., Maryland Heights; 2014 pp. 790–806.

[2] Amoah S, Boakye D, van Ree R, Yazdanbaksh M. Parasitic worms and allergies in childhood: Insights from population studies 2008–2013. Ped Allergy Immunol. 2014; 5: 208–217. 10.1111/pai.12174 24325393

[3] Lynch S, Wood R, Boushey H, Bacharier L, Bloomberg G, Kattan M, et al Effects of early-life exposure to allergens and bacteria on recurrent wheeze and atopy in urban children. J Allergy Clin Immunol. 2014; 134(3): 593–601. 10.1016/j.jaci.2014.04.018 24908147 PMC4151305

[4] McLoughlin R, Calatroni A, Visness C, Wallace P, Cruikshank W, Tuzova M, et al Longitudinal relationship of early life immunomodulatory T cell phenotype and function to development of allergic sensitization in an urban cohort. Clin Exp Allergy. 2011; 42: 392–404. 10.1111/j.1365-2222.2011.03882.x 22092655 PMC4162345

[5] Holt P, Rowe J, Kusel M, Parsons F, Hollams E, Bosco A, et al Towards improved prediction of risk for atopy and asthma amongst preschoolers: a prospective cohort study. J Allergy Clin Immunol. 2010; 125: 645–51. 10.1016/j.jaci.2009.12.018 20226300

[6] Harris JM, Williams HC, White C, Moffat S, Mills P, Newman Taylor AJ, et al Early allergen exposure and atopic eczema. Br J Dermatol. 2007; 156: 698–704. 17263823 10.1111/j.1365-2133.2006.07710.x

[7] Nwaru BI, Takkinen HM, Niemelä O, Kaila M, Erkkola M, Ahonen S, et al Introduction of complementary foods in infancy and atopic sensitization at the age of 5 years: timing and food diversity in a Finnish birth cohort. Allergy. 2013; 68: 507–16. 10.1111/all.12118 23510377

[8] Scott M, Roberts G, Kurukulaaratchy RJ, Matthews S, Nove A, Arshad SH. Multifaceted allergen avoidance during infancy reduces asthma during childhood with the effect persisting until age 18 years. Thorax. 2012; 67: 1046–51. 10.1136/thoraxjnl-2012-202150 22858926

[9] Strickland DH, Thomas JA, Mok D, Blank F, McKenna KL, Larcombe AN, et al Defective aeroallergen surveillance by airway mucosal dendritic cells as a determinant of risk for persistent airways hyperrepsonsiveness in experimental asthma. Mucosal Immunol. 2012; 5: 332–41. 10.1038/mi.2012.13 22354321

[10] Bjornsdottir S, Sigvaldadottir J, Brostrom H, Langvad B, Sigurdsson A. Summer eczema in exported Icelandic horses: influence of environmental and genetic factors. Acta Vet Scand. 2006; 48: 3–6. 16987399 10.1186/1751-0147-48-3 PMC1513129

[11] Sommer-Locher B, Endriss V, Fromm E. Various circumstances regarding initial allergen exposure and their influence on development of insect bite hypersensitivity in horses. J Equine Vet Sci. 2012; 32: 158–163.

[12] Schaffartzik A, Hamza E, Janda J, Crameri J, Marti E, Rhyner C. Equine insect bite hypersensitivity: What do we know?. Vet Immunol Immunopathol. 2012; 147: 113–126. 10.1016/j.vetimm.2012.03.017 22575371

[13] Siegal FP. Functional ontogeny of human lymphoid cells as a factor in maternal-foetaltolerance. Am J Reprod Immunol. 1981; 1 (2): 65–68. 6978080 10.1111/j.1600-0897.1981.tb00018.x

[14] Akdis M. Immune tolerance in allergy. Curr Opin Immunol. 2009; 21: 700–707. 10.1016/j.coi.2009.07.012 19700272

[15] Ryan C, Giguère S, Hagen C, Kalyuzhny A. Effect of age and mitogen on the frequency of interleukin-4 and interferon gamma secreting cells in foals and adult horses as assessed by an equine-specific ELISPOT assay. Vet Immunol Immunopathol. 2010; 133: 66–71. 10.1016/j.vetimm.2009.06.010 19616312

[16] Kachroo P, Ivanov I, Seabury A, Liu M, Chowdhay B, Cohen N. Age-related changes following in vitro stimulation with Rhodococcus equi of peripheral blood leucocytes from neonatal foals. PLOS One. 2013; 8(5): e62879 10.1371/journal.pone.0062879 23690962 PMC3656898

[17] Wagner B, Burton A, Ainsworth D. Interferon-gamma, interleukin-4 and interleukin-10 production by T helper cells reveals intact Th1 and regulatory Tr1 cell activation and a delay of the Th2 cell response in equine neonates and foals. Vet Res. 2010; 41: 47–61. 10.1051/vetres/2010019 20374696 PMC2865874

[18] Marti E, Gerber V, Wilson AD, Lavoie JP, Horohov D, Crameri R, et al Report of the 3rd Havemeyer workshop on allergic diseases of the Horse, Hólar, Iceland, June 2007. Vet Immunol Immunopathol. 2008; 126: 351–61. 10.1016/j.vetimm.2008.07.008 18775570

[19] Breathnach C, Sturgill-Wright T, Stiltner L, Adams A, Lunn DP, Horohov D. Foals are interferon gamma-deficient at birth. Vet Immunol Immunopathol. 2006; 112: 199–209. 16621024 10.1016/j.vetimm.2006.02.010

[20] Boyd NY, Cohen ND, Lim WS, Martens RJ, Chaffin MK, Ball M. Temporal changes in cytokine expression of foals during the first month of life. Vet Immunol Immunopathol. 2003; 92: 75–85. 12628765 10.1016/s0165-2427(03)00021-7

[21] Jacks S and Gigue`re S. Effects of inoculum size on cell-mediated and humoral immune responses of foals experimentally infected with Rhodococcus equi: a pilot study. Vet Immunol Immunopathol. 2010; 133: 282–286. 10.1016/j.vetimm.2009.08.004 19720402

[22] Jacks S, Gigue`re S, Prescott J. In vivo expression of and cell-mediated immune responses to the plasmid-encoded virulence-associated proteins of Rhodococcus equi in foals. Clin Vaccine Immunol. 2007; 14: 369–374. 17301216 10.1128/CVI.00448-06 PMC1865619

[23] Jacks S, Gigue`re S, Crawford P, Castleman W. Experimental infection of neonatal foals with Rhodococcus equi triggers adult-like gamma interferon induction. Clin Vaccine Immunol. 2007; 14: 669–677. 17409222 10.1128/CVI.00042-07 PMC1951072

[24] Sponseller B, de Macedo M, Clark S, Galup J, Jones D. Activation of peripheral Blood monocytes results in more robust production of IL-10 in neonatal foal compared to adult horses. Vet Immunol Immunopathol. 2009; 126: 351–61. 10.1016/j.vetimm.2008.09.013 18976818

[25] Hamza E, Steinbach F, Marti E. CD4(+)CD25(+) T cells expressing FoxP3 in Icelandic horses affected with insect bite hypersensitivity. Vet Immunol Immunopathol. 2012; 148: 139–44. 10.1016/j.vetimm.2011.05.033 21700344

[26] Hamza E, Akdis C, Wagner B, Steinbach F, Marti E. In vitro induction of functional allergen-specific CD4+CD25high Treg cells in horses affected with insect bite hypersensitivity. Clin Exp Allergy. 2013; 43: 889–901. 10.1111/cea.12131 23889243

[27] Hamza E, Doherr MG, Bertoni G, Jungi TW, Marti E. Modulation of allergy incidence in Icelandic horses is associated with a change in IL-4-producing T cells. Int Arch Allergy Immunol. 2007; 144: 325–337. 17671392 10.1159/000106459

[28] Hamza E, Gerber V, Steinbach F, Marti E. Equine CD4+CD25high T cells exhibit regulatory activity by close contact and cytokine-dependent mechanisms in vitro. Immunology. 2011; 134: 292–304. 10.1111/j.1365-2567.2011.03489.x 21977999 PMC3209569

[29] Billingham E, Brent L, Medawar P. Actively acquired tolerance to foreign cells. Nature. 1953; 172: 603–606. 13099277 10.1038/172603a0

[30] Brent L. The discovery of immunological tolerance. Hum Immunol. 1997; 52: 75–81. 9077556 10.1016/S0198-8859(96)00289-3

[31] Wang G, Miyahara Y, Guo Z, Khattar M, Stepkowski S, Wehano C. Default generation of neonatal regulatory T cells. The J Immuonol. 2010; 185: 71–78. 10.4049/jimmunol.0903806 20498359

[32] Sakaguchi S. Naturally arising CD4+ regulatory T cells for immunologic self-tolerance and negative control of immune responses. Annu Rev Immunol. 2004; 22: 531–62. 15032588 10.1146/annurev.immunol.21.120601.141122

[33] Mold J, Michaelsson J, Burt T, Muench M, Beckerman K, Busch M, et al Maternal alloantigens promote the development of tolerogenic foetal regulatory T cells in Utero. Science. 2008; 322: 1562–1565. 10.1126/science.1164511 19056990 PMC2648820

[34] Michaëlsson J, Mold J, McCune J, Nixon D. Regulation of T cell responses in the developing human fetus. J Immunol. 2006; 176: 5741–5748. 16670279 10.4049/jimmunol.176.10.5741

[35] Schaub B, Liu J, Schleich I, Hçppler S, Sattler C, von Mutius E. Impairment of T helper and T regulatory cell responses at birth. Allergy. 2008; 63: 1438–1447. 10.1111/j.1398-9995.2008.01685.x 18925880

[36] Fujimaki W, Takahashi N, Ohnuma K, Nagatsu M, Kurosawa H, Yoshida S, et al Comparative study of regulatory T cell function of human CD25+CD4+ T cells from Thymocytes, cord Blood, and adult peripheral blood. Clin Dev Immunol. 2008; 2008: 305–859. 10.1155/2008/305859 PMC2547481 18815628

[37] Ly NP, Ruiz-Perez B, McLoughlin RM, Visness CM, Wallace PK, Cruikshank WW, et al Characterization of regulatory T cells in urban newborns. Clin Mol Allergy. 2009; 7: 1–10. 10.1186/1476-7961-7-1 19586545 PMC2717905

[38] Yamagiwa S, Gray D, Hashimoto S, Horwitz D. A role for TGF-beta in the generation and expansion of CD4+CD25+ regulatory T cells from human peripheral blood. J Immunol. 2001; 166: 7282–7289. 11390478 10.4049/jimmunol.166.12.7282

[39] Zheng S, Wang J, Wang P, Gray D, Horwitz D. IL-2 is essential for TGF-beta to convert naive CD4^+^CD25^-^ cells to CD25^+^FoxP3^+^ regulatory T cells and for expansion of these cells. J Immunol. 2007; 178: 2018–2027. 17277105 10.4049/jimmunol.178.4.2018

[40] Liu R, Zhou Q, La Cava A, Campagnolo D, Van Kaer L, Dong Shi F. Expansion of regulatory T cells via IL-2 / anti-IL-2 mAb complexes suppresses experimental myasthenia. Eur J Immunol. 2010; 40: 1577–1589. 10.1002/eji.200939792 20352624 PMC3600978

