# Supplementary figures and images for: Regulatory T Cells in Early Life: Comparative Study of CD4+CD25high T Cells from Foals and Adult Horses
Source: PLoS One. 2015 Mar 19;10(3):e0120661. doi: 10.1371/journal.pone.0120661 (PMC4366079; doi:10.1371/journal.pone.0120661)

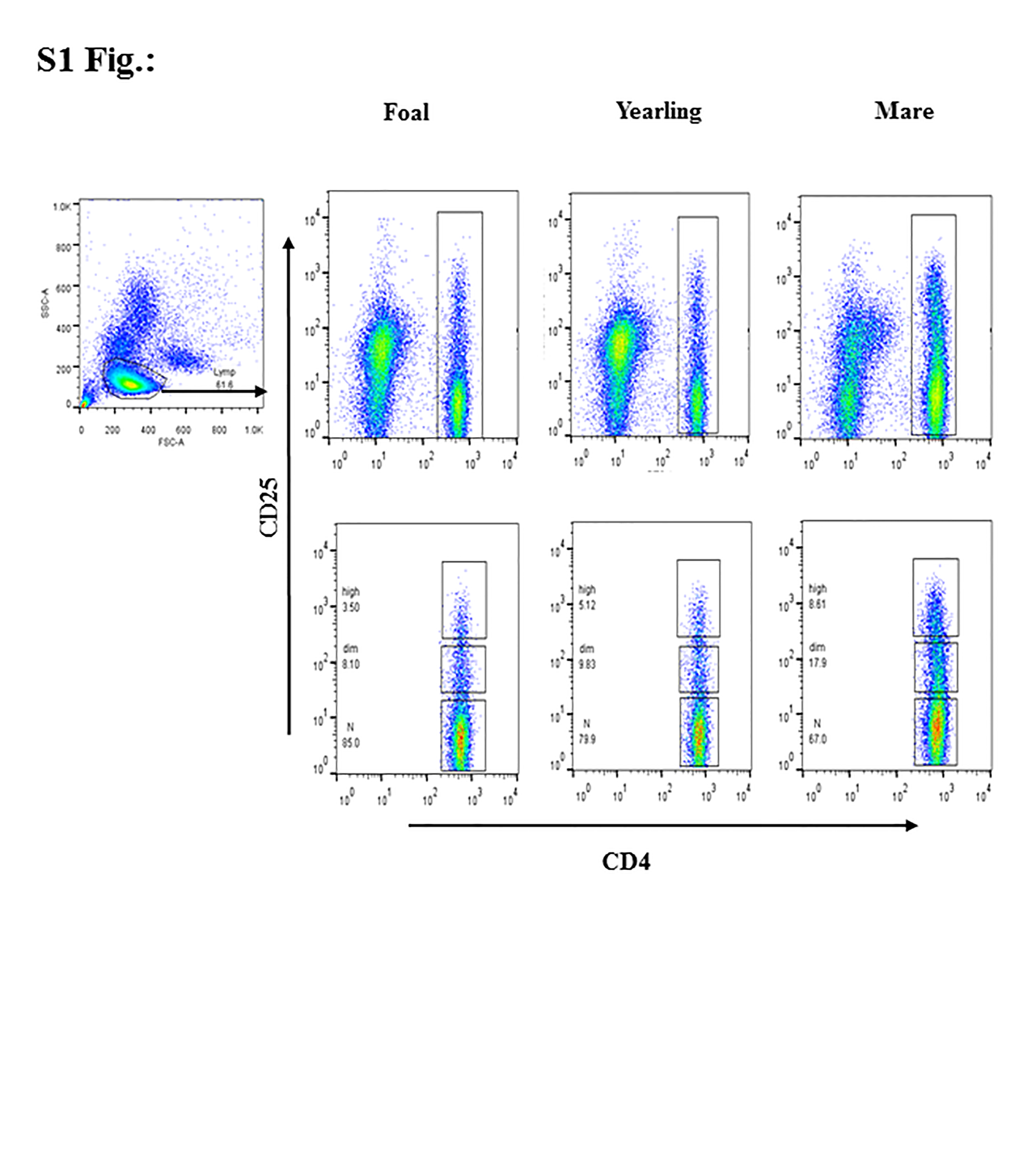

Supplement: S1 Fig — Freshly isolated peripheral blood mononuclear cells (PBMC) from foals, yearlings and mares were stained for CD4 and CD25. The relative amount of CD4+CD25− (N) CD4+CD25dim (dim) and CD4+CD25high (high) T cells was determined using flow cytometry as described before [28]. A gate was set around lymphocytes and gated cells were analysed for CD4 expression where another gate was positioned. CD4+ cells were subsequently analysed for CD25 expression. CD25− cells were first distinguished from those with a dim fluorescence signal (dim) and additionally from those with a distinct (>10 fold) brighter fluorescence signal (high). Subsequently, three gates with small gaps between them were placed, defining the three CD4+ subpopulations. (TIF) [file pone.0120661.s001.tif]

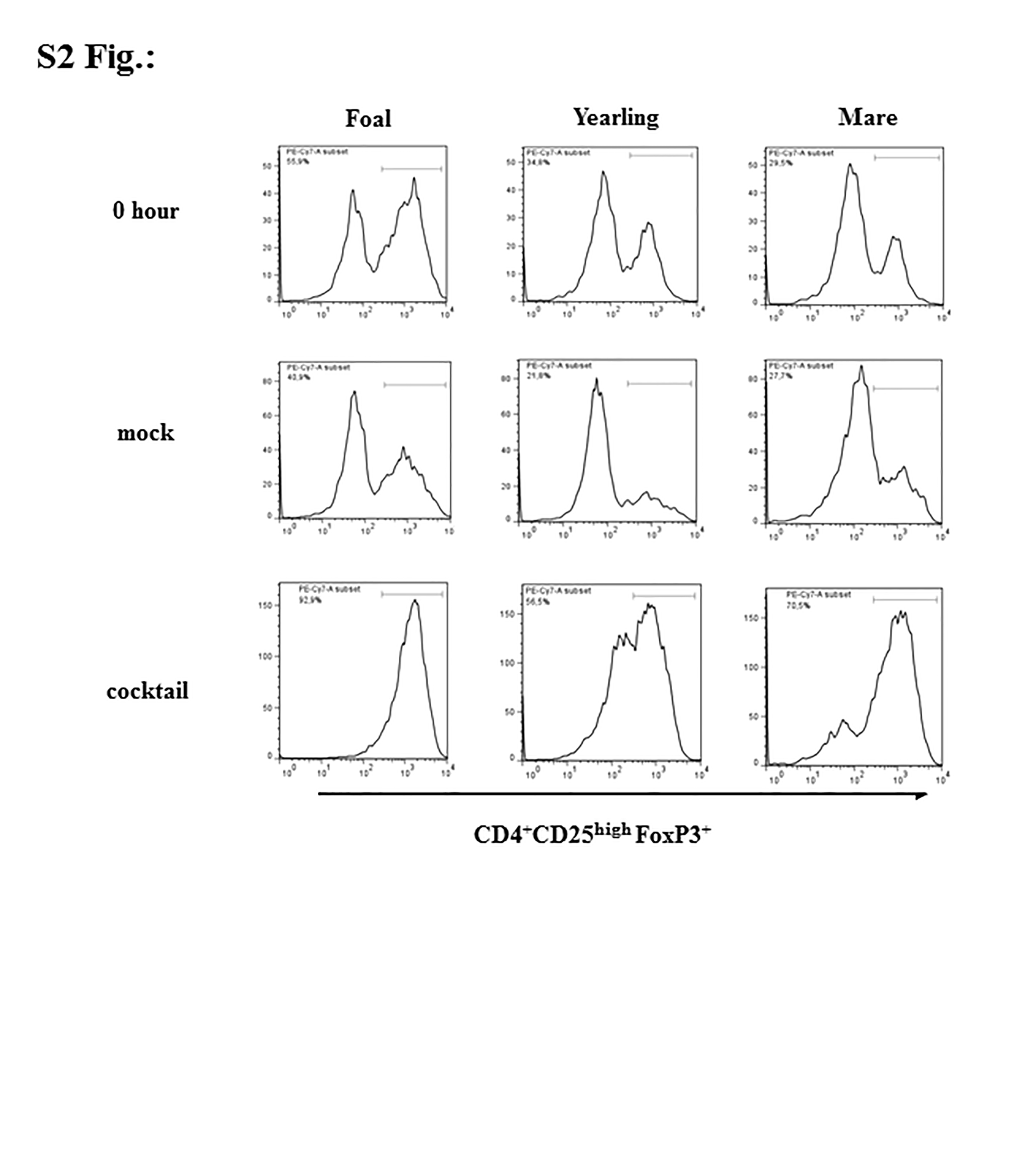

Supplement: S2 Fig — PBMC freshly isolated (0 h) or cultured for 4 days without (mock) or with the cocktail (cocktail) were stained for CD4, CD25 and FoxP3. The expression of FoxP3 within CD4+CD25high cells is presented. Horses included are foals, yearlings and mares. (TIF) [file pone.0120661.s002.tif]

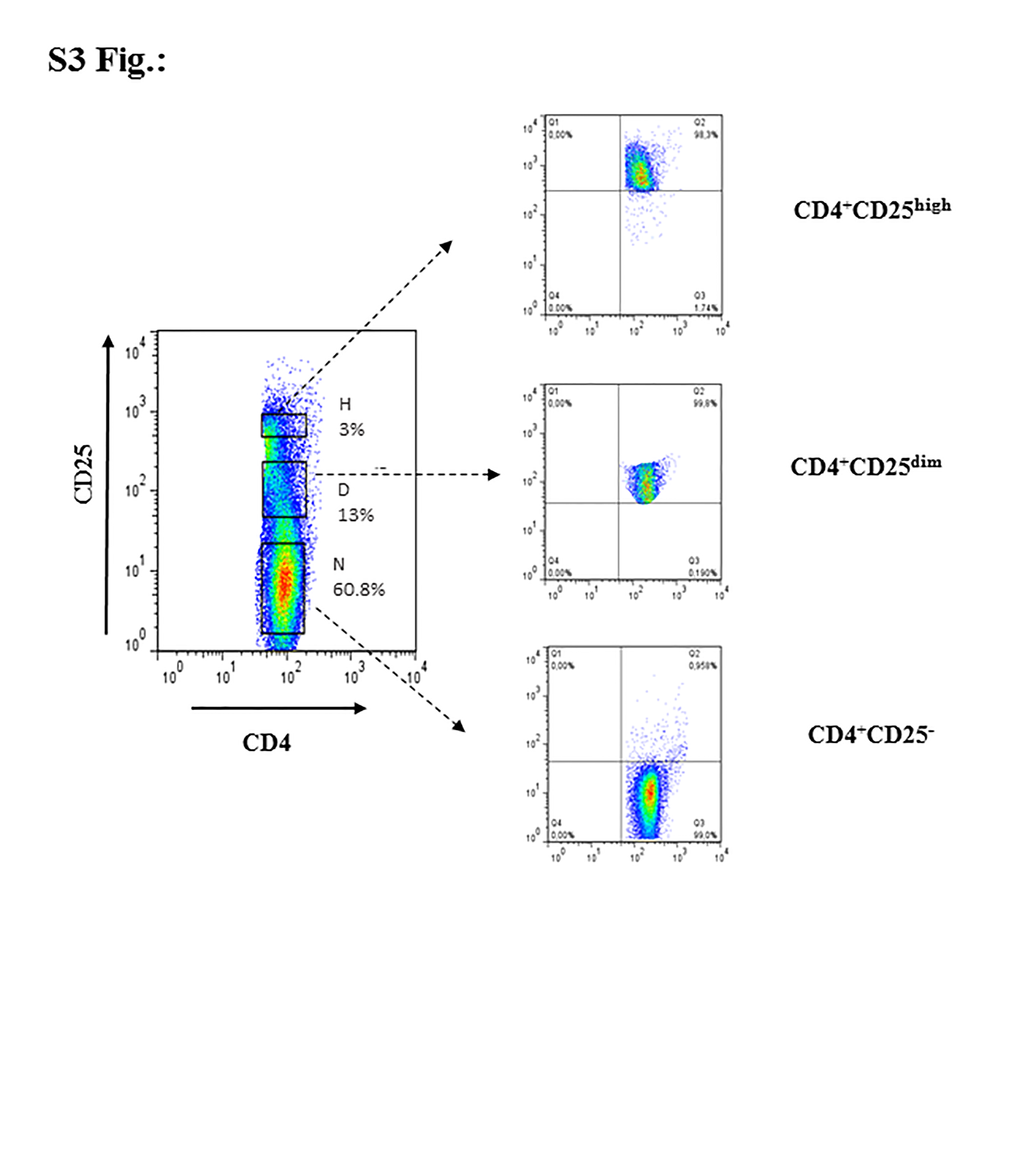

Supplement: S3 Fig — After isolation of PBMC, CD4+ cells were enriched by positive magnetic cell separation (MACS). The CD4+ cells were stained for CD25 as described [28]. CD4+CD25−, CD4+CD25dim and CD4+CD25high T cells were identified and sorted as previously [28], using a more stringent gating than for phenotyping (S1 Fig.) to avoid cross contamination. Analysis of the three subpopulations after sorting, demonstrated > 98% purity for each of the three subpopulations. (TIF) [file pone.0120661.s003.tif]

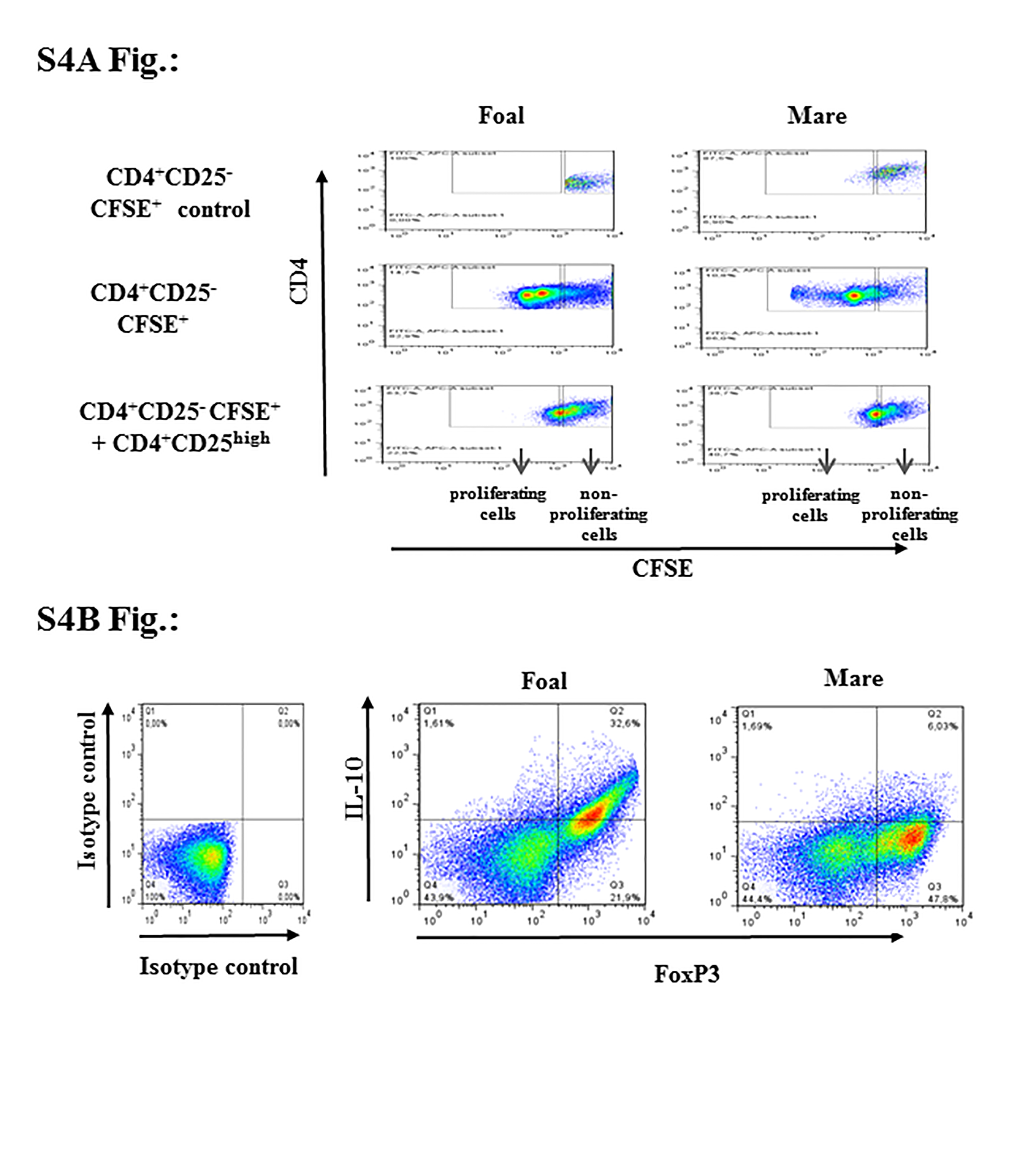

Supplement: S4 Fig — CD4+CD25− lymphocytes sorted from freshly isolated PBMC of foals and mares as described in S3 Fig. were labelled with carboxyfluorescein diacetate succinimidyl ester (CFSE) and cultured without (CD4 + CD25 − control, top row) or with irradiated allogenic PBMC alone (CD4 + CD25 −, middle row) or in the presence of sorted CD4+CD25high (CD4 + CD25 − + CD4 + CD25 high, bottom row) cells. After 4 days, the cells were harvested and stained for FoxP3 and IL-10 or the relevant isotype controls. Analysis was performed using Flowjo software. A) The gated CFSE-labelled CD4+CD25− cells were analysed for percentage proliferation by setting gates for proliferating (FITC-A, APC-A Subset) and non-proliferating (FITC-A, APC-A Subset-1) cells. B) The percentages of single positive IL-10 +FoxP3− (Q1), double positive IL-10 + FoxP3 + (Q2) and single positive FoxP3 +IL-10− (Q3) within CD4+CD25high cells were measured by flow cytometry. (TIF) [file pone.0120661.s004.tif]

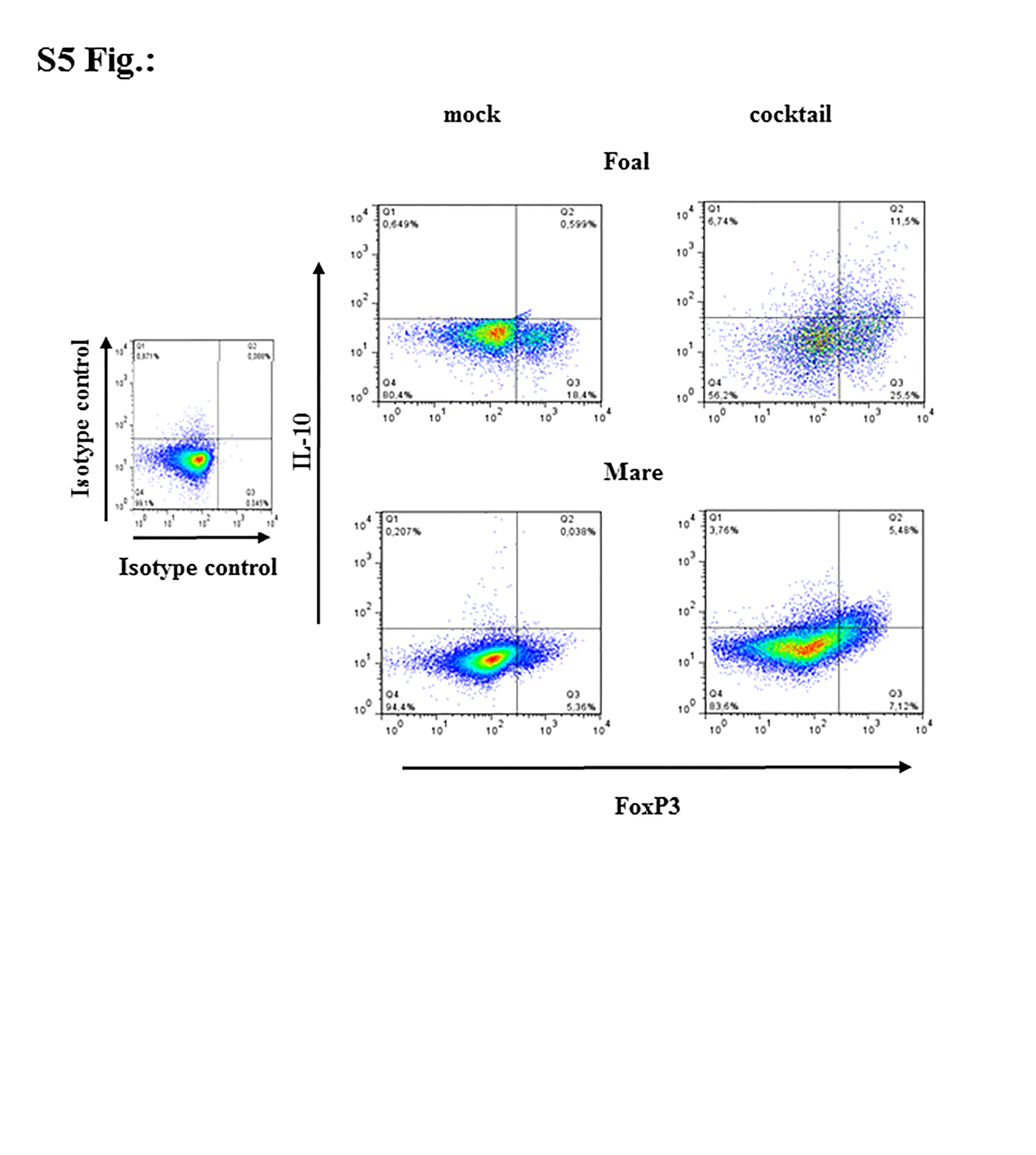

Supplement: S5 Fig — CD4+CD25high lymphocytes sorted from freshly isolated PBMC of foals and mares were left either un-stimulated (mock) or stimulated with cocktail. After 4 days, the cells were harvested and stained for FoxP3 and IL-10. The percentages of single positive IL-10 +FoxP3− (Q1), double positive IL-10 + FoxP3 + (Q2) and single positive FoxP3 +IL-10− (Q3) were measured by flow cytometry. (TIF) [file pone.0120661.s005.tif]

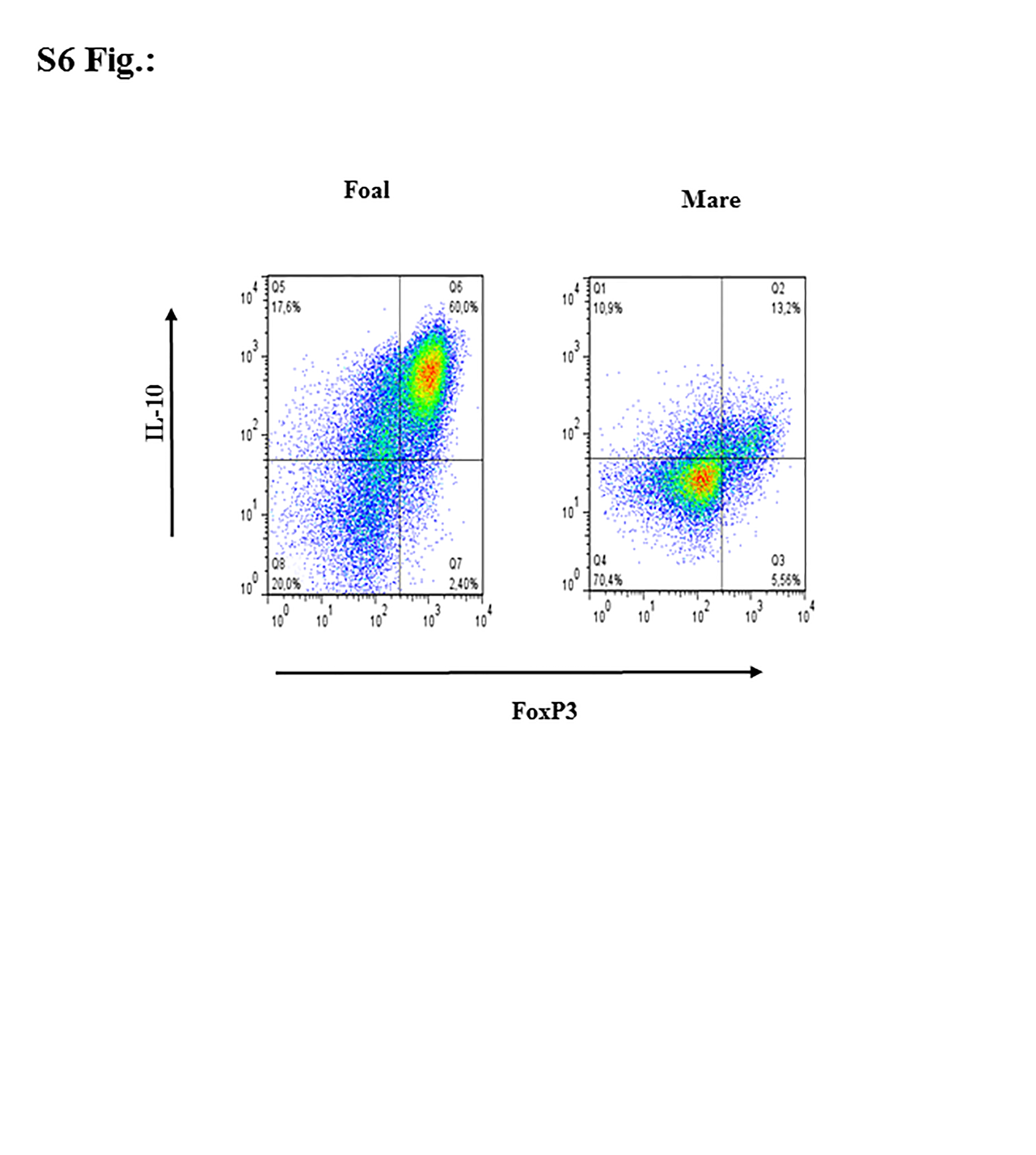

Supplement: S6 Fig — CD4+CD25− lymphocytes sorted from freshly isolated PBMC of foals and mares were cultured with the cocktail for four days, harvested, stained for CD25 and resorted for induced CD4+CD25high (I CD4 + CD25 high) cells. The I CD4 + CD25 high cells were stained for FoxP3 and IL-10. The percentages of single positive IL-10 +FoxP3− (Q1), double positive IL-10 + FoxP3 + (Q2) and single positive FoxP3 +IL-10− (Q3) were measured. (TIF) [file pone.0120661.s006.tif]
